# Editorial: Antioxidant potential of polyphenolic and flavonoid compounds

**DOI:** 10.3389/fchem.2024.1463755

**Published:** 2024-07-31

**Authors:** Abdur Rauf, Zubair Ahmad, Dorota Formanowicz, Giovanni Ribaudo, Taghrid S. Alomar

**Affiliations:** ^1^ Department of Chemistry, University of Swabi, Swabi, Pakistan; ^2^ Chair and Department of Medical Chemistry and Laboratory Medicine, Poznan University of Medical Sciences, Poznań, Poland; ^3^ Department of Molecular and Translational Medicine, University of Brescia, Brescia, Italy; ^4^ Department of Chemistry, College of Science, Princess Nourah Bint Abdulrahman University, Riyadh, Saudi Arabia

**Keywords:** antioxidant, polyphenolic, flavonoid, anti-inflammatory, medicinal plants

In ancient times and in the modern era, natural antioxidants have been known to play a vital role in promoting health and managing critical disease conditions (Gupta and Sharma, 2006). Plant-derived compounds like polyphenols and flavonoids possess remarkable antioxidant potential. These molecules have evolved in plants to counteract oxidative stress, and when consumed, they can confer similar benefits to humans (Bautista-Hernández et al., 2021).

This Research Topic, “Antioxidant Potential of Polyphenolic and Flavonoid Compounds,” collected four studies that investigate the antioxidant properties of various plant species, including *Dodonaea viscosa* Jacq., *Habenaria aitchisonii* Reichb., *Viola canescens* Wall, and the enzymatic secretome of *Botrytis cinerea*. These studies demonstrate the significant antioxidant, anti-inflammatory, and antinociceptive activities of these plant-derived compounds, both *in vitro* and *in vivo, and further testify to the international scientific community’s interest in investigating* beneficial natural compounds.

The article entitled “*In vitro* and *in vivo* antioxidant therapeutic evaluation of phytochemicals from different parts of *Dodonaea viscosa* Jacq” contributes significantly to the expanding index of natural medicines by meticulously exploring the antioxidant potential of *D. viscosa* Jacq. (Plant-DV) (Khan et al.). Antioxidants are crucial in scavenging free radicals, thereby preventing cellular damage and contributing to the prevention and treatment of various diseases. In their study, the authors employed a comprehensive approach to evaluate the antioxidant properties of aqueous extracts from the leaves and flower-containing seeds of Plant-DV. By using both *in vitro* and *in vivo* methodologies, the researchers provide a holistic view of the antioxidant capabilities of this plant, underscoring its potential therapeutic applications (Dhaniya and Parihar, 2019). More into detail, the investigation involved a series of assays to measure glutathione (GSH) levels, hydrogen peroxide (H_2_O_2_) scavenging effects, glutathione-S-transferase (GST) activity, and catalase (CAT) activity. Furthermore, the *in vivo* assessments performed on Wistar Albino rats, using vitamin C as a positive control, provided a solid comparative basis for evaluating the therapeutic potential of Plant-DV. Key findings from the study reveal that the flower-containing seeds exhibit higher GSH content compared to the leaves in both normal and oxidatively stressed conditions. Notably, the leaves demonstrated superior H_2_O_2_ scavenging effects. Overall, the results indicate that different parts of Plant-DV possess distinct antioxidant profiles, and the non-toxic nature of the plant extracts, even at high doses, highlights their safety for potential therapeutic use. The observed increases in GSH, GST, and CAT levels in the liver and kidneys of treated animals further reinforce the therapeutic promise of Plant-DV. These findings align with the broader body of research emphasizing the importance of natural antioxidants in managing oxidative stress-related diseases, including cancer, cardiovascular diseases, and neurodegenerative disorders. By demonstrating significant antioxidant activities both *in vitro* and *in vivo*, this study positions *D. viscosa* Jacq. as a promising candidate for developing natural antioxidant therapies. In conclusion, it substantially contributes to the natural antioxidant research, paving the way for novel therapeutic approaches. As we continue to seek effective and safe treatments for oxidative stress-related conditions, the findings of this study underscore the enduring relevance and potential of natural medicines in modern healthcare (Riaz et al., 2012).

Exploring plant-derived compounds has been an enduring pursuit in the quest for novel therapeutic agents (Chaachouay and Zidane, 2024). The research article “Evaluation of *Habenaria aitchisonii* Reichb. for antioxidant, anti-inflammatory, and antinociceptive effects with *in vivo* and *in silico* approaches” presents a comprehensive examination of *Habenaria aitchisonii* Reichb., shedding light on its promising bioactive properties (Asiri et al.). The study commenced with the phytochemical analysis of *H. aitchisonii* using GC-MS, identifying 18 distinct compounds. The identified compounds, particularly germacrone, highlight the plant’s potential as a source of significant bioactive molecules. The robust chemical profiling lays a solid foundation for subsequent bioactivity assays. More into detail, the *in vitro* assays revealed the potent antioxidant and anti-inflammatory activities of the chloroform (Ha.Chf) and ethyl acetate (Ha.EtAc) fractions of *H. aitchisonii*. These fractions exhibited substantial inhibition in ABTS, DPPH, and H_2_O_2_ scavenging assays with Ha.Chf showing the highest activity. The COX-2 and 5-LOX inhibition assays further affirmed the anti-inflammatory potential of these fractions, showcasing their effectiveness comparable to standard drugs like celecoxib and montelukast. *In vivo* studies demonstrated that H. aitchisonii is safe at oral doses of up to 3,000 mg/kg, indicating a favorable toxicity profile. The antinociceptive effects, evaluated through acetic acid-induced writhing, formalin, and hot plate tests, revealed significant analgesic properties of Ha.Chf and Ha.EtAc fractions. These findings suggest both peripheral and central antinociceptive mechanisms, further reinforcing the therapeutic potential of *H. aitchisonii* in pain management. The carrageenan-induced paw edema model and phlogistic agents assays provided insights into the anti-inflammatory mechanisms, highlighting the inhibition of COX-2 and 5-LOX pathways by Ha.Chf. The study also evaluated the antioxidant effects *in vivo*, showing that H. aitchisonii significantly ameliorates LPS-induced oxidative stress by enhancing levels of GSH, SOD, and CAT while reducing MDA levels. These antioxidant properties further support its therapeutic potential in inflammatory conditions. Complementing the experimental findings, *in silico* docking studies provided a molecular perspective on the interaction of identified compounds with the COX-2 protein. Compounds 1, 2, 8, and 11 demonstrated strong calculated binding affinities, corroborating the experimental anti-inflammatory results and suggesting specific molecular targets for the bioactive compounds in *H. aitchisonii*. This multifaceted study of *Habenaria aitchisonii* Reichb demonstrates its significant antioxidant, anti-inflammatory, and antinociceptive activities, both *in vitro* and *in vivo*. Integrating chemical profiling, bioactivity assays, and computational docking provides a comprehensive understanding of its therapeutic potential. These findings validate the traditional use of H. aitchisonii in folk medicine and pave the way for further research and development of plant-derived therapeutic agents. The promising results of this study warrant additional investigations into the clinical applications of *H. aitchisonii*, potentially contributing to the development of new treatments for inflammatory and pain-related conditions.

In the pursuit of identifying potent natural antioxidants, the study “Antioxidant Potential of Alkaloids and Polyphenols of *Viola canescens* Wall Using *In Vitro* and In Silico Approaches” offers a profound exploration into the bioactive properties of *Viola canescens*. This research further underlines the significance of ethnomedicinal plants in traditional healthcare and their potential in modern therapeutic applications. The investigation began with extracting various fractions from the crude methanolic extract of *Viola canescens*. The antioxidant potential of these fractions was meticulously evaluated using Tetraoxomolybdate (VI) and DPPH assays, which are renowned for their reliability in assessing antioxidant activity. The results revealed that the aqueous extract of *V. canescens* exhibited noteworthy antioxidant and free radical scavenging activities, with the crude flavonoid extract showing significant inhibition of cell growth at an IC_50_ value of 57.863 μg/mL. The research further examines the molecular mechanisms underlying these activities through *in silico* docking studies and molecular dynamic simulations. These computational approaches validated the *in vitro* findings and provided a deeper understanding of the molecular mechanisms involved. The docking studies, for instance, highlighted a strong interaction between emetine and the aromatase protein, suggesting emetine’s potential role as a natural antioxidant. Moreover, the study’s results demonstrated a clear dose-response relationship, where higher concentrations of the extracts corresponded to greater antioxidant activity. Among the fractions tested, crude flavonoid extract (CTF), ethyl acetate fraction (EAF), and aqueous fraction (AqF) stood out for their superior antioxidant potential, likely due to their rich polyphenolic content. The DPPH radical scavenging assay further corroborated these findings, showcasing the scavenging ability of the extracts. This assay confirmed that crude alkaloids and flavonoids were particularly effective in neutralizing free radicals. A significant aspect of this study is the integration of molecular docking analysis, which revealed that emetine, quercetin, and violanthin, the essential phytochemicals in *V. canescens*, exhibited strong calculated binding affinities with critical enzymes like the progesterone receptor and aromatase. These interactions were further substantiated by molecular dynamic simulations, which confirmed the stability and efficacy of these ligand-receptor complexes. Additionally, the study employed Molinspiration to evaluate the drug-likeness of these phytochemicals, confirming their suitability as potential therapeutic agents. Except for violanthin, all compounds adhered to Lipinski’s rule of five, indicating favorable pharmacokinetic properties. In conclusion, this comprehensive study not only reinforces the traditional uses of *V. canescens* in treating various ailments but also highlights its potential as a rich source of natural antioxidants and bioactive molecules. The findings open new avenues for pharmaceutical applications, particularly in combating oxidative stress-related diseases such as cancer. This study serves as a testament to the invaluable role of ethnomedicinal plants in advancing modern healthcare (Ahmad et al.).

Phenoxy radical coupling reactions are pivotal for the natural synthesis of complex molecules like lignin, and they offer substantial potential for laboratory synthesis of high-value polyphenolic compounds (Zetzsche et al., 2022). In the study titled “Study of phenoxy radical couplings using the enzymatic secretome of *Botrytis cinerea*,” the authors examine the enigmatic behavior of phenoxy radicals, aiming to control and harness these reactions for the synthesis of specific dimers and tetramers of phenylpropanoid and stilbene derivatives. Building on previous research where stilbenes and phenylpropanoids were dimerized using the enzymatic secretome of *B. cinerea*, this study investigates the influence of different structural features on radical coupling efficiency. A crucial discovery is the role of an exocyclic conjugated double bond in facilitating efficient radical reactions. The formation of phenylpropanoid trimers and tetramers, through decarboxylation that regenerates this reactive moiety, was meticulously examined. The study outlines a stepwise approach to monitor and control the radical coupling reactions. The researchers achieved esterification to form ethyl ferulate, starting with ferulic acid, followed by enzymatic conversion to a dimer. Subsequent saponification and decarboxylation yielded poacic acid, which produced specific dimeric compounds upon further enzymatic treatment. This method allowed selective synthesis of high-purity phenylpropanoid derivatives, showcasing the potential for controlled radical coupling in polyphenol synthesis. The results emphasize the significance of structural features in achieving efficient radical coupling, particularly the presence of a para-phenol conjugated to an exocyclic double bond. Compounds lacking this feature exhibited variable reactivity, highlighting the complexity of these reactions. The study also explored the behavior of various phenolic substrates, noting that while some compounds like dihydro-resveratrol formed dimers, others, such as 4-O-methyl-resveratrol, did not undergo dimerization, underscoring the nuanced influence of molecular structure on reactivity. The findings open a path for further exploration of radical coupling mechanisms, potentially leading to the development of new methods for synthesizing complex polyphenolic structures. In particular, by understanding and controlling these reactions, researchers can expand the repertoire of synthesized polyphenolic compounds, with implications for pharmaceuticals, materials science, and beyond. In fact, this work not only contributes to the fundamental understanding of radical chemistry but also offers practical methodologies for synthesizing valuable polyphenolic derivatives (Huber et al.).

In conclusion, it can be stated that this Research Topic substantially contributes to the field of natural antioxidant research, highlighting the significance of plant-derived compounds in promoting health and managing disease conditions, with particular attention to mechanistic investigations. The studies presented here offer valuable insights that could pave the way for novel therapeutic approaches, and their findings warrant further investigation into the clinical applications of these plant-derived compounds.
